# Cost-benefit relation of diet and probiotics in iatrogenic bowel irregularity (IBI)

**DOI:** 10.3389/fphar.2014.00014

**Published:** 2014-02-17

**Authors:** Eric Claassen

**Affiliations:** ^1^Department of Virology, Erasmus Medical CenterRotterdam, Netherlands; ^2^Athena Institute, Vrije UniversiteitAmsterdam, Netherlands

**Keywords:** probiotic, prevention, bowel, irregularity, antibiotic-associated diarrhea (AAD), *Clostridium difficile*-associated diarrhea (CDAD)

## Introduction

In this issue Lenoir-Wijnkoop et al. (2014) show that an affordable and widely available fermented dairy drink containing a known and well-documented probiotic could lead to health care system cost savings. This paper is just one in a surprisingly large number of very recent papers on probiotics, antibiotic-associated diarrhea (AAD), and *Clostridium difficile*-associated diarrhea (CDAD, recurrent-CDAD, or CD infection: CDI). The revival of attention for CDI is mainly caused by the emergence of new hyper virulent strains, a decrease of clinical response to antibiotics, an increase of incidence and prevalence and the existence of at least three different toxins. *Clostridium difficile* has a broad clinical manifestation from asymptomatic (contagious) carriers, via diarrhea toward pseudomembranous colitis, toxic megacolon, and death. Since *Clostridium difficile* is a spore former it can still cause endogenous self-contamination even (or especially) after antibiotic treatment. Furthermore, these spores are a contamination hazard in health care facilities and they heighten the transmission rates (*R*-value). Taken together, all these factors (reviewed in Hell et al., 2013) result in a high unmet medical need with significant socio-economic impact. Here, we would like to put both the clinical and economic impact of this paper in a broader “state of the art” context, by adding alternative new insights and references not yet available to the Lenoir-Wijnkoop paper.

## Cost

The total cost of AAD, CDAD, and CDI is dependent on the calculation method used, as discussed at length in the Lenoir-Wijnkoop paper, but is in the order of several tens of billions of dollars annually. Direct cost of recurrent CDAD/CDI ranges from 2000 (USA) to 3000 (UK) euro per case/year with health care system costs in the range of 4–6000 euro's peaking as high as 11,000 euro for recurrent-CDI (reviewed in Hell et al., 2013). The added, and real, cost to society is much higher. Use of “double” doses probiotics in Canada (where 1 in 10 CDI patients succumbs to the disease) resulted in cost savings of up to a 1000 euro per patient (*n* = 1000 patients; Fansi et al., 2012).

## Re-poop-ulation with donor feces

Repoopulation, a term originally coined by Petrof et al. (2013) based on their astonishing results, pertains to the re-balancing of an aberrant microbiota by infusing donor-feces. In a very recent double blind placebo controlled study (RCT), recurrent CDI could be solved in over 80% of investigated cases by duodenal infusion of donor-feces, where vancomycin resolved disease in 31% and vancomycin in combination with bowel lavage in only 23%. These results were so dramatic that the RCT was stopped, after independent review in an interim analysis, in order to provide feces transplantation to the placebo group (Van Nood et al., 2013). Feces transplantation is under discussion with the FDA for presumed safety issues (but still allowed for recurrent CDI) and remains a costly procedure. The high price and even a ban on repoopulation has also led to (unwise and unwanted) do it yourself repoopulation instruction videos on the internet. Clearly, reintroducing a complete new microbiota is a long way from managing the same problem with a fermented dairy drink with one to ten selected strains of lactic acid bacteria.

## Unmet medical need

AAD and CDAD are only two of several forms of bowel irregularity introduced by medical intervention. Other iatrogenic bowel irregularities (IBI) are introduced for instance by: enteral-nutrition-deficiencies, hospital diet, colonoscopy-damage/perforation, trauma, stress, therapeutic irradiation, colectomy-associated—pouchitis or—short-bowel-syndrome, surgery, chemotherapy, morphine and other medicines, hospital infections, and the length of time people are bedridden (general lack of exercise). These factors can deregulate normal gut motility and gut function. Moreover, they can influence the living microbiota (10^14^ bacteria) of the gut. However, it should be stressed that most of these IBI are usually mild and self-limiting, but in some cases this can lead to persistent or recurrent obstipation or diarrhea, prolonged hospital stay, serious complications, and death (Hell et al., 2013). The incidence of CDAD and CDI varies greatly from center to center and has an enormous impact on the outcome of the study. When event rates are much lower than predicted, the resulting low absolute risk reduction might interfere with significance even in large and overpowered studies (Daneman, 2013). So there is an enormous center-to-center variation in CD-associated problems and when incidence of CDAD is lower than 1.2% probiotics might not be cost effective, as recently shown in a meta-analysis based on all available data (Daneman, 2013). However, recently it was also shown that in a 240 bed hospital over the course of 22 months almost two thousand patients were treated for a (presumed) *C. dif* issue. Eventually, only 15% (!) had lab confirmed positive tests (unpublished, see Wickline, 2013). Consequently, from premature babies with NEC (necrotizing enterocolitis) via healthy adults with weight (Mekkes et al., 2013) or bowel problems, right through to the elderly, bowel irregularities (in need of bona fide diagnostics) cause significant morbidity and mortality and form a largely unmet medical need throughout even a relatively “healthy” life (Islam et al., 2012; Weenen et al., 2013a).

## Diet, probiotics, and prebiotics

Prebiotics are non-digestible food ingredients that stimulate the growth and/or activity of bacteria in the digestive system in ways claimed to be beneficial to health (i.e., linked to stimulation of probiotics). They were first identified and named by Marcel Roberfroid in 1995 and are also known as oligosaccharides or soluble-fibers (not to be confused with food fiber). Human milk oligosaccharides (HMOS) represent the third most abundant component of human breast milk. More than a hundred structurally distinct HMOS have been identified. Recent studies have started to link individual HMOS to infant health and disease and suggest the importance of a mother-infant match with regard to HMOS composition (Marx et al., 2014). Milk from domestic mammals has 10–100 fold less oligosaccharides than human milk (Table 1). Oligosaccharides like galacto-(GOS) and fructo-oligosaccharides (FOS, including inulin) are routinely added to most infant formulas and are also available in powdered form. It has long been known that diets supplemented with GOS and FOS have a prebiotic effect, encouraging the growth of our “endogenous probiotics” like bifidobacteria and reducing opportunistic pathogens like *Escherichia coli*, *Bacteroides* spp, and *Clostridium* spp. Barrett (2013). Paradoxically, prebiotics should not be added to probiotics during GI transit because they cause a switch from logarithmic to stationary growth phase (Golowczyc et al., 2013) which lowers their resistance to gastric acid (low pH) and bile, by lowering the expression of stress proteins (personal communication Dr. K. Venema). On the other hand, metabolizable sugars (like glucose) provide ATP via glycolysis thereby enabling proton exclusion potentially enhancing survival during gastric transit (Corcoran et al., 2005). Next to these indirect prebiotic effects in the gut, GOS, and FOS also have direct effects, such as pathogen-adhesion-inhibition to the gut epithelium and modulating binding of selectins (Barrett, 2013; Roberts et al., 2013). Consequently taking probiotics with small amounts of sugar helps the strains to survive gastric and bile juices. In the diet, the vegetables that contain relatively large amounts of oligosaccharides (like inulin) are, strangely enough, not the ones we use when experiencing IBI (iatrogenic bowel irregularities) like e.g., diarrhea. Furthermore, the first eight vegetables in the list are routinely found in a Mediterranean diet. Hospital dieticians and cooks could use the data of the Lenoir-Wijnkoop study in conjunction with high GOS and FOS foods for synergy (known as synbiotics) and enhance the calculated beneficial effects by minor dietary interventions at marginal extra cost.

**Table 1:** **Prebiotics, (a.k.a. soluble fibers) improving growth of probiotic bacteria, as found in dietary components and milk ranked by quantitative availability**.

**Recovered oligosaccharides as % weight/weight fresh**	
Chicory/endive stalks	15–20
Garlic	9–16
Black salsify and leek	4–10
Onion and artichoke	2–6
Asparagus	2–3
Wheat	1–4
Oats (and human breast milk)	0.5–1.5
Banana	0.3–0.7
Domestic-placental-mammal milk	0.01–0.1

## Food-pharma convergence and regulations

As we described recently, industries are converging at the Food-Pharma interface and work in different regulatory and reimbursement areas on either functional foods or medical foods (Weenen et al., 2013b). Remarkably, although the core technology domain is food within the medical-food industry the technological development is mainly driven by pharmaceutical/pharmacological technologies. When classified as functional foods, probiotic effects pertaining to human health, consist of claims subject to evaluation by regulatory institutes such as the European Food Safety Authority (EFSA) Panel on Dietetic Products, Nutrition and Allergies (NDA). As recently reviewed 78% of all analyzed health claims are considered by the NDA Panel as (possibly) beneficial to human health, in particular the gut health effects. However, in many cases, the scientific substantiation of a particular health claim, when analyzed in a Pharma like manner, was deemed insufficient, and most applications were turned down (Binnendijk and Rijkers, 2013). Currently, there is a vivid debate as to whether functional foods should be indeed regulated more stringently as medical foods (as EFSA is now doing), the largely EU-policy-driven outcome is eagerly awaited by scientists, clinicians and industry alike (Hill and Sanders, 2013). For near-future health claim applications concerning probiotics to be successful, they should include precise dose and strain specifications, specific statements on what exactly the microorganism affects, and overpowered intent to treat (ITT) analysis of the clinical trials in the targeted (general) population. An excellent and recent example of how such study should be performed along those very stringent guidelines, convincingly showing a dose-dependent 50% reduction of AAD with a probiotic mixture was published by Ouwehand et al. (2014).

## Safety

Recently we systematically studied adverse events as related to probiotics, in all placebo controlled RCT's those AEs found were considered unrelated to the study product (which was also well-tolerated), and there were no major safety concerns in either verum or placebo. However, inconsistent, imprecise, and potentially incomplete reporting as well as the variation in probiotic strains, dosages, administration regimes, study populations and reported outcomes, greatly limits the generalizability of conclusions and argues convincingly for obligatory, and standardized behavior on adverse events (CTCAE) reporting in “food” studies (Van den Nieuwboer et al., 2014). The safety, quality, and quantity (CFU), specifically in relation to antibiotic sensitivity, of medicinal products containing probiotics also needs to be established as recently and satisfactorily done for Polish products (Wiatrzyk et al., 2013).

## Dose and product choice

Without going into too much detail it is clear from literature that probiotics work in a dose-dependent manner with effectiveness for AAD or CDAD at or around 10–17 billion bacteria per day (e.g., double dose in Lenoir-Wijnkoop study and Ouwehand et al., 2014). Most products on the market do not contain those high numbers. However, the dosing is strain-dependent and dose-response relations need to be established for each different strain/product or cocktail/combination (Hell et al., 2013). Moreover, recently Douillard et al. (2013) showed that the draft genomes of two competing brands of dairy drink based probiotic products are close to identical to each other and differ by no more than minor chromosomal re-arrangements, substitutions, insertions, and deletions, as evident from the verified presence of one insertion-deletion (InDel) and only 29 single-nucleotide polymorphisms (SNPs). Their observations could imply that results obtained in human experiments performed with the Actimel^®^ (as used in the Lenoir-Wijnkoop study) and Yakult^®^ strains can be compared with each other as these strains share a recent common ancestor. Nevertheless, as the human intervention trials are not conducted on the strains only but mainly on the final food form (matrix/sugar/additives), in accordance with the current regulation, and therefore differences in matrix, the bacterial composition and metabolites can lead to significant differences in outcomes. Similar relations could exist for other, competing products in the market and accentuate the EFSA call for full genome sequencing identification data and clear and detailed description of matrix and formulation. Finally, dose and product choice also has an impact when comparing single- and multi species/strain products. As recently shown in the Allen study (cfr Daneman, 2013) not all strain/species/dose combinations will perform effectively. However, this and other negative studies have barely any impact on the meta-analysis outcome of the accepted effectiveness of (working) probiotics in AAD (Cochrane review by Goldenberg et al., 2013). Furthermore, such negative studies are less convincing when only low incidence (and consequently low absolute risk reduction) of CDAD or AAD are found in the control group (Daneman, 2013).

## Conclusions

As described in the Lenoir-Wijnkoop paper in this issue a new and effective generation of probiotic products, in which all aspects of health, disease, and pathogen-exclusion are incorporated, is emerging (cfr also: Hell et al., 2013; Ouwehand et al., 2014). When given orally and in the right dose in combination with exercise and an appropriate diet, probiotics can give a clear healthcare system cost reduction for several forms of IBI with notable examples like AAD, CDAD, and CDI. Probiotics are safe and reduce disease/antibiotic-related adverse events by at least 20%. Treatment dose and protocols are product- and strain-dependent. Studies combining biological, clinical, and economic data help predict whether the benefits of a protocol or policy (e.g., probiotics and diet) outweigh its costs. Perfect evaluation of all present and future costs and benefits is challenging, but ranking of best alternatives should result in economic efficiency and social welfare in vulnerable patient groups.
